# Immune activation correlates with and predicts CXCR4 co-receptor tropism switch in HIV-1 infection

**DOI:** 10.1038/s41598-020-71699-z

**Published:** 2020-09-28

**Authors:** Bridgette J. Connell, Lucas E. Hermans, Annemarie M. J. Wensing, Ingrid Schellens, Pauline J. Schipper, Petra M. van Ham, Dorien T. C. M. de Jong, Sigrid Otto, Tholakele Mathe, Robert Moraba, José A. M. Borghans, Maria A. Papathanasopoulos, Zita Kruize, Francois W. D. Venter, Neeltje A. Kootstra, Hugo Tempelman, Kiki Tesselaar, Monique Nijhuis

**Affiliations:** 1 Department of Medical Microbiology, Virology, University Medical Center Utrecht (UMCU), Utrecht, The Netherlands; 2 Ezintsha, Faculty of Health Sciences, University of the Witwatersrand, Johannesburg, South Africa; 3 Ndlovu Research Consortium, Elandsdoorn, Limpopo Province South Africa; 4 Center for Translational Immunology, UMCU, Utrecht, The Netherlands; 5 HIV Pathogenesis Research Unit, Faculty of Health Sciences, University of the Witwatersrand, Johannesburg, South Africa; 6 Amsterdam University Medical Center, Amsterdam Infection and Immunity Institute, Academic Medical Center of the University of Amsterdam, Amsterdam, The Netherlands

**Keywords:** Infectious diseases, Immunology

## Abstract

HIV-1 cell entry is mediated by binding to the CD4-receptor and chemokine co-receptors CCR5 (R5) or CXCR4 (X4). R5-tropic viruses are predominantly detected during early infection. A switch to X4-tropism often occurs during the course of infection. X4-tropism switching is strongly associated with accelerated disease progression and jeopardizes CCR5-based HIV-1 cure strategies. It is unclear whether host immunological factors play a causative role in tropism switching. We investigated the relationship between immunological factors and X4-tropism in a cross-sectional study in HIV-1 subtype C (HIV-1C)-infected patients and in a longitudinal HIV-1 subtype B (HIV-1B) seroconverter cohort. Principal component analysis identified a cluster of immunological markers (%HLA-DR^+^ CD4^+^ T-cells, %CD38^+^HLA-DR^+^ CD4^+^ T-cells, %CD38^+^HLA-DR^+^ CD8^+^ T-cells, %CD70^+^ CD4^+^ T-cells, %CD169^+^ monocytes, and absolute CD4^+^ T-cell count) in HIV-1C patients that was independently associated with X4-tropism (aOR 1.044, 95% CI 1.003–1.087, p = 0.0392). Analysis of individual cluster contributors revealed strong correlations of two markers of T-cell activation (%HLA-DR^+^ CD4^+^ T-cells, %HLA-DR^+^CD38^+^ CD4^+^ T-cells) with X4-tropism, both in HIV-1C patients (p = 0.01;p = 0.03) and HIV-1B patients (p = 0.0003;p = 0.0001). Follow-up data from HIV-1B patients subsequently revealed that T-cell activation precedes and independently predicts X4-tropism switching (aHR 1.186, 95% CI 1.065–1.321, p = 0.002), providing novel insights into HIV-1 pathogenesis and CCR5-based curative strategies.

## Introduction

The identification of chemokine receptors as co-receptors for human immunodeficiency virus 1 (HIV-1) infection has enhanced our understanding of cellular entry, viral transmission, and pathogenesis of infection^1^ and has played a pivotal role in the ongoing development of HIV-1 cure strategies^2,3^. HIV-1 transmission almost exclusively involves CCR5 (R5)-tropic founder viruses, which dominate during early infection^4–7^.

Years after transmission, viruses also able to use the CXCR4 (X4) co-receptor (X4-tropic viruses) are observed as the dominant plasma population in 50% of individuals infected with HIV-1 subtype B (HIV-1B) who are untreated or experiencing virological failure^4,5,8,9^. The percentages of X4-tropic viruses in the HIV-1 subtype C (HIV-1C) population are currently poorly defined in treatment naive and treatment experienced individuals^2,10–12^. This switch from R5- to X4-tropism is associated with accelerated CD4^+^ T-lymphocyte depletion and disease progression^8,9,13^. Furthermore, it prohibits the use of CCR5 as a target for antiretroviral therapy^14–16^ and cure strategies^17–20^.

The viral determinants of HIV-1 co-receptor tropism map largely to the region encoding the V3-loop of the viral envelope (gp120 surface glycoprotein)^21^ and genetic tropism tests (GTT) allow computational prediction of co-receptor tropism based on the sequence of this region (WebPSSM^22^ and Geno2Pheno^23^). Since relatively few genetic changes are required to obtain X4-utilisation, it is paradoxical that the switch from R5- to X4-tropism does not occur more rapidly and frequently in vivo. This suggests that *only* viral genetic changes are not sufficient and host (immunological) factors are required to create an environment in which X4-tropic viral strains can emerge as the dominant viral population. While it remains largely unknown which conditions facilitate a tropism switch, presence of X4-tropic virus is associated with accelerated CD4^+^ T-cell loss and progression to AIDS^24^. Given the predictive value of host immune activation for this immunological decline^25–28^, we hypothesize that immune activation may be the driving force behind co-receptor switching.

Therefore, we set out to explore associations between X4-tropism and host immunological factors in a cross-sectional study of HIV-1C and HIV-1B patients. We subsequently investigated whether immune activation measured during early chronic infection can predict the subsequent occurrence of a switch from R5- to X4-tropism in a unique historic HIV-1B seroconverter cohort.

## Results

### Cross-sectional HIV1-C study

To explore correlations between host immunological markers and X4-tropism, we measured an array of markers and performed a GTT in 100 South African HIV-1 C infected ART-naive patients who enrolled in a cross-sectional study. The median age of all enrolled participants was 37 years [interquartile range (IQR): 31–40], they had a median CD4^+^ T-lymphocyte count (CD4 count) of 215 cells/µL (IQR: 54–412) and 61% were female (61/100) (Table 1). HIV-1 was predicted to be X4-tropic in 24% of participants using a False-Positive Rate (FPR) of ≤ 3.5% (Table 1).

**Table 1:** Clinical characteristics and co-receptor tropism in HIV-1C cohort.

Variables	Overall	CCR5 (FPR > 3.5)	CXCR4 (FPR ≤ 3.5)	p-value
*N*	*100*	*76*	*24*	*N/A*
Gender *(female)*	61% (61)	62% (47)	58% (14)	*0.95*
Age *(years)*	36.6 [31.3–40.4]	36.1 [31.1–40.6]	37.8 [33.5–40.1]	*0.48*
CD4 count (*cells/µL*)	215 [54–412]	229 [81–435]	46 [15–386]	**0.02**
TB status (*TB-diagnosed*)	21% (16/77)	18% (12/65)	33% (4/12)	*0.26*
Lowest FPR	26.2 (26.4)	34.2 (25.5)	1.1 (1.1)	*N/A*

Units and non-significant p-values are displayed in italics. p-values below 0.05 are displayed in bold. Data displayed as mean (standard deviation), mean percentage (absolute number), or as median [interquartile range]. Due to 23 patients with no conclusive outcome of TB assessment, definitive TB status was only available for 77 cases. Significance testing for TB status was performed using Fisher Exact testing due to low case numbers.

*N* number, *FPR* false-positive rate, *TB* tuberculosis.

In univariate analysis, X4-tropism was positively associated with the following cellular markers of immune activation: %HLA-DR^+^ CD4^+^ T-cells (aOR 1.12, 95% CI 0.912–1.36, p = 0.01) and %CD38^+^HLA-DR^+^ CD4^+^ T-cells (aOR 0.99, 95% CI 0.79–1.22, p = 0.03) (Table 2). Negative associations were seen between X4-tropism prediction and CD4 count (aOR 0.89, 95% CI 0.80–0.99, p = 0.02; Table 2).

**Table 2:** Cross-sectional HIV-1C analysis: correlates of viral co-receptor tropism.

Variable	Summary estimates			
	Mean (standard deviation)		p-value	aOR [95% CI]
	CCR5-tropic (FPR > 3.5)	CXCR4-tropic (FPR ≤ 3.5)		
Markers of immune activation				
CD4+ T cell count *(cells/µl)*^a^	199 (225)	126 (216)	**0.02**	0.89 [0.80–0.99]
CD8+ T cell count (^*10*^*log cells/µl)*^a^	119 (156)	158 (105)	0.11	*NA*
CD38+ *(% in CD4+ T cells)*	58.5 (17.4)	62.0 (14.7)	0.87	*NA*
HLA-DR+ *(% in CD4+ T cells)*^a^	14.4 (14.6)	26.6 (25.4)	**0.01**	1.12 [0.91–1.36]
HLA-DR+ CD38+ *(% in CD4+ T cells)*^a^	7.3 (6.1)	12.0 (16.8)	**0.03**	0.99 [0.79–1.22]
CD70 *(% in CD4+ T cells)*^a^	1.1 (2.1)	2.0 (6.0)	0.08	0.98 [0.86–1.11]
HLA-DR+CD38+ *(% in CD8+ T cells)*^a^	22.7 (13.3)	15.6 (14.7)	0.52	*NA*
CD57+ *(% in CD4+ T cells)*	45.6 (16.7)	39.3 (14.3)	0.41	*NA*
CD169 *(% in classical monocytes)*	93.0 (35.5)	96.6 (20.1)	0.05	0.97 [0.84–1.11]
Central memory T cell *(% of CD4+ T cells)*	27.8 (13.4)	26.6 (10.0)	0.95	*NA*
TNFR2 *(pg/ml)*^a^	1,931 (1,226)	1,806 (809)	0.24	*NA*
sCD163 *(pg/ml)*^a^	383.4 (7,527.7)	34.6 (1,924.2)	0.49	*NA*
Receptor-ligand interactions				
CCR5+ CXCR4+ *(% in CD4+ T cells)*^a^	5.5 (17.8)	6.4 (34.6)	0.20	*NA*
CCL3 (MIP-1⍺) *(pg/ml)*^a^	103.9 (116.1)	103.6 (72.6)	0.91	*NA*
CCL4 (MIP-1β) *(pg/ml)*^a^	57.3 (45.4)	65.1 (37.0)	0.95	*NA*
CCL5 (RANTES) *(pg/ml)*^a^	30,445 (95,015)	35,019 (179,839)	0.17	*NA*
CXCL12 (SDF-1⍺) *(pg/ml)*^a^	35.5 (824.8)	88.7 (2,014.2)	0.82	*NA*
CXCL10 (IP-10) *(pg/ml)*^a^	510.3 (816.3)	556.3 (259.1)	0.68	*NA*
IL-7 *(pg/ml)*^a^	117.3 (52.7)	106.9 (91.7)	0.92	*NA*

Units are displayed in italics.

*aOR* adjusted odds ratio, *FPR* false-positive rate, *95% CI* 95% confidence interval.

P-values below 0.05 are displayed in bold.

^a^Data was log-transformed, means are log-corrected, standard deviation is original. p-value derived from unpaired t-test of (log-transformed) data.

**Figure 1:**
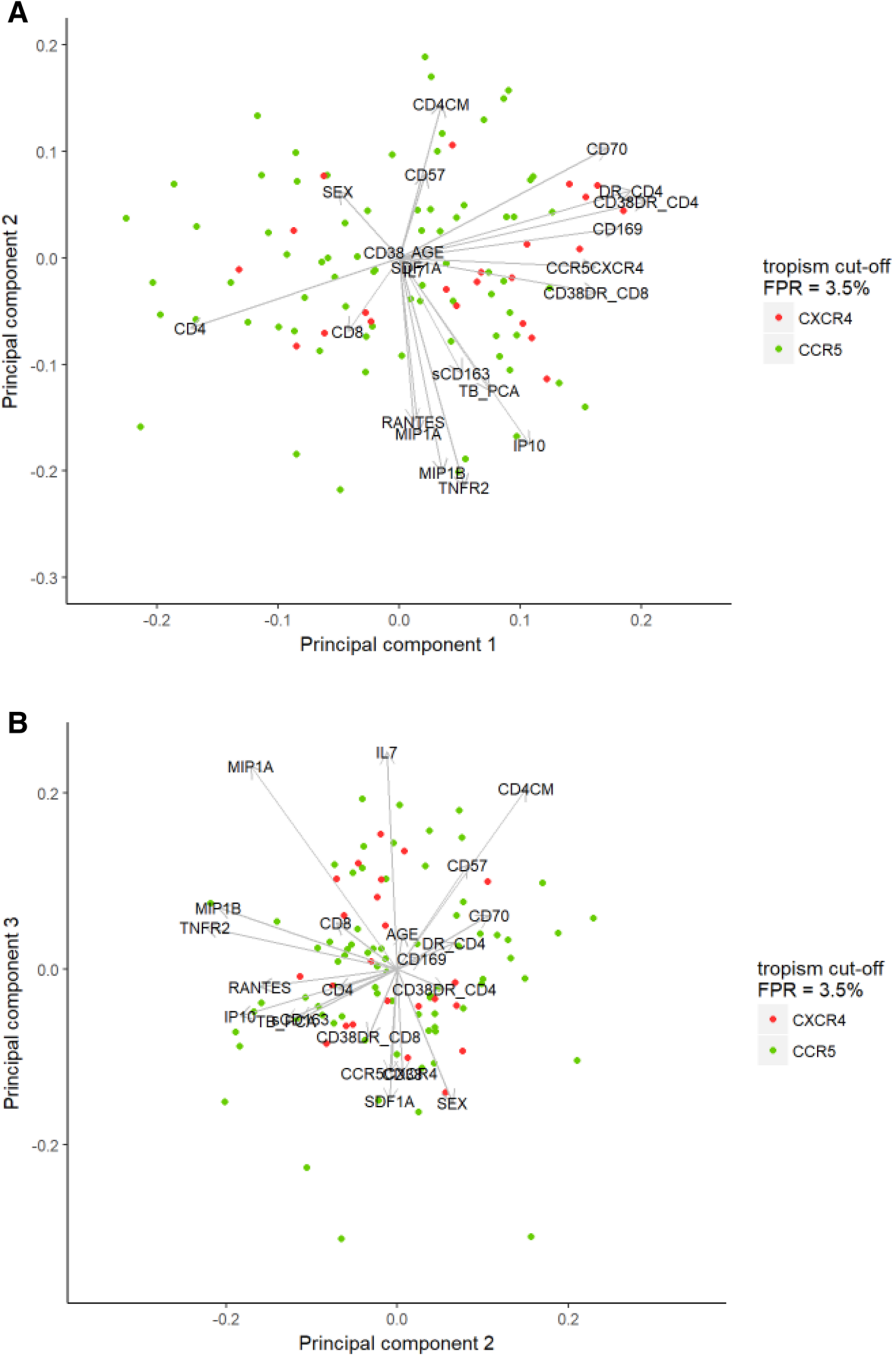
**A** Variable loadings and individual observations for principal components 1 and 2. **B:** Variable loadings and individual observations for principal components 2 and 3.

Subsequently, we performed principal component analysis in order to assess the complex interdependencies between immunological markers and their association with co-receptor tropism. Dominant contributors to PC1 included CD4 count, all cellular markers of immune activation that showed univariate correlations to X4-tropism (%HLA-DR^+^ CD4^+^ T-cells, %CD38^+^HLA-DR^+^ CD4^+^ T-cells), as well as several other cellular immune activation markers (%CD38^+^HLA-DR^+^ CD8^+^ T-cells, %CD70^+^ CD4^+^ T-cells and %CD169^+^ monocytes). Cellular immune activation markers correlated positively to PC1, and CD4 count correlated inversely to PC1, reflecting ongoing immune activation and CD4^+^ T-cell depletion respectively. Dominant contributors to PC2 included soluble markers of immune activation (TNFR2, IP-10) and ligands of CCR5 (MIP-1α, MIP-1β, and RANTES), and all were inversely correlated to PC2 (Supplementary Table 2). In line with the results of the univariate analysis, some separation of X4-tropism was observed along PC1 reflecting cellular immune activation and CD4 depletion (Fig. 1). Multivariable logistic regression of the first four PCs revealed that PC1 showed a significant positive correlation with X4-tropism [aOR 1.044 (95% CI 1.003–1.087), p = 0.0392], while other PCs did not show significant correlations and did not improve the model in stepwise backward variable selection.

In order to verify the robustness of the PCA and influence of the outlying data points, k-fold cross-validation of the PCA and multivariable analysis of the resulting PCs was performed (k = 20, iterations = 100), yielding 2000 subsets each containing 95% of patients in the original dataset. Results of PCA iterations on each subset revealed that CD4 count, %HLA-DR^+^ CD4^+^ T-cells, %HLA-DR^+^CD38^+^ CD4^+^ T-cells and %HLA-DR^+^D38^+^ CD8^+^ T-cells were all major contributors (absolute rotation ≥ 0.3) to PC1 in > 95% of cases, indicating high robustness of PC1. Results of the multivariable analyses on each iteration of the PCA results showed that the correlation between PC1 and X4-tropism was consistently detected and had a p-value of < 0.05 in 41.3%.

### Cross-sectional and longitudinal HIV-1B study

Subsequently, we investigated the association between T-cell activation and co-receptor switch and its causal relationship in a unique historic HIV-1B infected ART-naive seroconverter cohort. All 82 study participants were included prior to HIV-1B seroconversion, and followed-up for a median duration of 2520 days (IQR 1883–3614) after seroconversion (Table 3). All participants were male and had a median age of 37 years (IQR: 31–41) at seroconversion. At one year after seroconversion, median CD4 count was 605 cells/µL (IQR: 450–813), and median log viral load was 4.5 ^10^log copies/mL (IQR: 3.9–4.8). A phenotypically detected switch from R5-tropic to X4-tropic virus during follow-up was observed in 36.6% (30/82) of patients at a median of 1410 days (IQR: 874–1967) after seroconversion. These patients were at a markedly increased risk of developing AIDS and death (Table 3).

**Table 3:** Clinical characteristics and co-receptor tropism in HIV-1B cohort.

Variables	Overall	Remains CCR5-tropic (NSI-virus)	switches to CXCR4-tropic (SI-virus)	p-value
*n*	*82*	*52*	*30*	*N/A*
Gender (*male*)	100% (82)	100% (52)	100% (30)	*N/A*
Age (*years*) at seroconversion	36.6 [31.3–40.7]	36.6 [32.8–42.4]	36.8 [30.2–39.0]	*0.338*
CD4+ T-cell count (*cells/µL*) at 1 year after seroconversion	605 [450–813]	610 [490–823]	570 [408–773]	*0.482*
HIV-RNA load (^*10*^*log* *copies/mL*) at 1 year after seroconversion	4.5 [3.9–4.8]	4.4 [3.9–4.8]	4.5 [4.2–5.0]	*0.192*
AIDS diagnosis^a^	53.7% (44)	40.4% (21)	76.7% (23)	**0.003**
Death^a^	41.5% (34)	28.8% (15)	63.3% (19)	**0.003**

Data displayed as percentage (absolute number) or as median [interquartile range].

*n* number, *SI* syncytium-inducing, *NSI* non-SI.

Units and non-significant p-values are displayed in italics. p-values below 0.05 are displayed in bold.

^a^Occuring during follow-up of median 2520 days [IQR: 1883–3614] after seroconversion.

At five years of follow-up, 36.6% (30/82) had experienced a phenotypically detected switch to X4-tropic virus. A cross-sectional analysis was first performed at this timepoint to replicate the findings made in the analysis of HIV-1C infected patients. Univariate analysis confirmed the negative association between X4-tropism and CD4 count (p = 0.004) and the positive association between X4-tropism and %HLA-DR^+^ CD4^+^ T-cells (p = 0.0003) and %HLA-DR^+^CD38^+^ CD4^+^ T-cells (p = 0.0001) at five years of follow-up. We subsequently performed Cox proportional hazard analysis of longitudinal data to assess the effect of markers of cellular immune activation measured at one year after seroconversion on subsequent tropism switching. Four cases were excluded from this analysis. In these cases, switch to X4-tropic virus was observed within the first year after seroconversion, prior to first measurement of immunological marker data.

In the univariate Cox proportional hazard analysis of 78 included patients, CD4 count and viral load measured at one year after seroconversion were not significantly associated with a subsequent switch from R5- to X4-tropic virus (Table 3). However, simultaneously measured levels of cellular immune activation markers that dominantly contributed to X4-tropism in both cross-sectional studies (%HLA-DR^+^ CD4^+^ T-cells, %HLA-DR^+^ CD38^+^ CD4^+^ T-cells) were positively associated with subsequent switch to X4-tropism (Table 4). In a multivariable Cox proportional hazard analysis %HLA-DR^+^ CD4^+^ T-cells measured at one year after seroconversion remained significantly associated with subsequent switch to X4-tropic virus. However, at this time point early after seroconversion, no significant association was observed between age, CD4 count, HIV-RNA load and future coreceptor switch (Table 4) in this HIV-1B cohort, despite the observation of a significant negative correlation between CD4 count and X4 tropism seen in the HIV-1C cohort. Additional immunological markers measured 5 years post seroconversion in the HIV-1B cohort can be found in supplemental Table 3.

**Table 4:** Longitudinal HIV-1B analysis: predictors of switch of co-receptor tropism.

Variable	Median [IQR]		HR [95% CI]	aHR [95% CI]
	Remains CCR5-tropic (NSI virus)	Switches to CXCR4-tropic (SI visus)		
Age (*years*) at seroconversion	36.6 [32.8–42.4]	36.8 [30.2–40.0]	0.976^(ns)^ [0.920–1.035]	0.962^(ns)^ [0.909–1.019]
HIV-RNA load (^*10*^*log* *copies/mL*) at 1 year after seroconversion	4.4 [3.9–4.8]	4.5 [4.2–5.0]	1.572^(ns)^ [0.891–2.775]	1.521^(ns)^ [0.859–2.692]
CD4+ T-cell count (*cells/µL*) at 1 year after seroconversion	610 [490–823]	570 [408–773]	0.959^(ns)^ [0.885–1.040]	1.009^(ns)^ [0.920–1.106]
%HLA-DR+ in CD4+ T-cells at 1 year after seroconversion	4.5 [3.5–6.8]	6.0 [4.8–10.6]	1.174** [1.062–1.298]	1.186** [1.065–1.321]
%HLA-DR+ CD38+ in CD4+ T-cells at 1 year after seroconversion	3.0 [2.1–4.2]	3.3 [2.7–6.4]	1.210** [1.070–1.369]	–

Four patients were excluded from the analysis who had evidence of a switch before the first year post seroconvcersion, 16 out of 78 samples were X4 tropic in this analysis.

%HLA-DR+CD38+ CD4+ T-cells was not incorporated in the multivariable analysis due to observed collinearity with %HLA-DR+ CD4 T-cells (Spearman’s ρ = 0.94).

*(a)HR* (adjusted) Hazard ratio, *SI* syncytium-inducing, *NSI* non-SI, *95% CI* 95% confidence interval.

(ns) = p ≥ 0.05, * = p < 0.05, ** = p ≥ 0.01.

## Discussion

Selection of HIV-1 variants that can use the CXCR4 co-receptor for viral entry is associated with accelerated disease progression and jeopardizes HIV-1 cure strategies specifically targeting CCR5-tropic HIV-1. However, whether these X4-tropic viruses are the cause or the consequence of disease progression remains unknown. In two cross-sectional studies we have identified markers of CD4^+^ T-cell activation that correlated with the selection of X4-tropic HIV-1. Analysis of longitudinal data from an HIV-1B seroconverter cohort showed that higher levels of these CD4^+^ T-cell activation markers in early infection precede and independently predict subsequent emergence of X4-tropic viral strains. To our knowledge, this is the first report demonstrating this causal relationship, suggesting that X4-tropic viruses are not the cause of immune activation and disease progression but should merely be seen as a consequence of ongoing immune activation. 

We observed that CD4^+^ T-cell activation predicts the emergence of X4-tropic viral strains and hypothesize that this is through modulation of host target cell availability. In early infection, the CD4^+^ memory T-cell is the primary HIV-1 target cell. The relatively high cell surface expression of CCR5 compared to CXCR4 on these cells and the higher affinity for CD4 of R5-tropic versus X4-tropic viruses creates selective pressure towards R5-tropic over X4-tropic virus in early infection^29,30^. As the transition of R5- to X4-tropism enables targeting of naive CD4^+^ T-cells that predominantly express CXCR4, depletion of the memory T-cell pool over time is thought to confer an evolutionary advantage to X4-tropic virus^29^. It has been firmly established that the setpoint of HIV-1-associated immune activation in the early stages of chronic infection is closely linked to the rate of T-cell turnover and subsequent CD4^+^ T-cell depletion^25–28^. We therefore hypothesize that immune activation resulting in memory CD4^+^ T-cell depletion may function as the driver of eventual outgrowth of X4-tropic HIV-1, which are favoring the remaining population of naive T-cells.

Our hypothesis is in line with a study demonstrating that selection of X4-tropic viruses before initiation of antiretroviral therapy was associated with only partial immune recovery during treatment^31^. A possible confounder in this study was the generally lower baseline CD4 count in the immunological non-responder group. However, some patients with low CD4 counts (< 150 cells/µl) belonged to the immune responders, whereas others with a baseline count of  > 150 cells/µl had a CD4 increase < 400 cells^31^. In the HIV-1C cohort, there was a strong association between lower CD4 count and X4-tropism at the time of tropism determination. In the HIV-1B cohort, CD4 count within one year after seroconversion was not significantly associated with subsequent development of X4-tropism.

In addition to host target cell availability, other immunological drivers of co-receptor tropism have been proposed. The upregulation of CXCR4 co-receptor expression by IL7 and TNF-receptor II would facilitate the targeting of CXCR4 expressing cells^32–35^. Differential presence of the natural ligand of CXCR4, SDF-1α (CXCL12), that inhibits CXCR4 binding by X4-tropic virus, has also been suggested as a potential host factor. CXCL12 and its production by dendritic cells potentially explains why the transfer of X4-variants to CXCR4 co-receptors typically does not predominate during early infection^36,37^. In addition, it has been proposed that X4-tropic virus is more sensitive to neutralization by host antibodies compared to its R5-tropic counterpart virus^38^. While we cannot definitively exclude that these and other mechanisms may also play a role, our extensive exploratory analysis in HIV-1C incorporated most of these markers and did not reveal any significant associations with co-receptor tropism.

While this study sheds light on the influence of immune activation levels on HIV-1 co-receptor tropism and takes into account a large array of other potential covariates, by nature of its observational design, it does not directly address the underlying biological mechanism. On the other hand, an important strength of this study is the insight gained from two distinct treatment naive patient populations including a longitudinal analysis in a unique historic cohort of HIV-1B infected patients in The Netherlands from an era prior to the availability of treatment, enabling study of the natural course of infection. Currently, effects of ART on HIV co-receptor tropism are not fully understood but several studies indicate preferential reduction of X4 viruses during therapy^31,39^.

Most knowledge on X4-tropic HIV-1 stems from research on HIV-1B infected individuals from developed countries, which account for only 10% of infections worldwide. The data regarding co-receptor switching in HIV-1C infection, which represents over 50% of all HIV-1 infections worldwide is conflicting^40^. Prevalence estimates vary widely and are likely subject to patient selection^41–49^. We and others have previously shown that contrary to some reports, R5-tropic to X4-tropic co-receptor switching in HIV-1C infected patients in developing countries occurs regularly^10,12^. The 24% prevalence of infection with X4-tropic HIV-1 encountered in the current cross-sectional study is largely in line with these reports.

Our observation that higher levels of immune activation can predict the subsequent selection of X4-tropic viral variants as the dominant viral population clearly demonstrated that X4-tropism is not the favorable viral phenotype in the immunocompetent host. This implies that in the immunocompetent host X4-tropic viral variants may be frequently generated and be present as a minority population that can rapidly expand if the CCR5-tropic viral variants are inhibited by for instance CCR5-based therapy or CCR5-based curative strategies. We and others have indeed shown the rapid outgrowth of a pre-existing X4-tropic viral population in the presence of maraviroc therapy^50,51^ that immediately reverts after discontinuation of the CCR5 inhibitor^16^. Furthermore, we have shown rapid replacement of R5-tropic strains by a pre-existing X4-tropic minority variant in a patient receiving an allogeneic transplant with stem cells lacking expression of the CCR5 co-receptor (CCR5∆32)^52^. Taken together, X4-tropic viruses may be present in an HIV-1 infected individual but will only represent a minority population until replication of R5-tropic viruses is specifically inhibited or the host immune system is deteriorated, allowing an selective advantage of X4-tropic virus. These data highlight that a deeper understanding of the correlates of HIV co-receptor tropism is vitally important and that in-depth co-receptor analyses are essential for future CCR5-based cure strategies such as stem cell transplantation and gene therapy.

## Methods

### Subjects and study design

#### Cross-sectional HIV-1C study

Recruitment was performed at the Ndlovu Medical Centre, a rural clinic situated in Dennilton, Sekhukune district, Limpopo province, South Africa, providing comprehensive medical service including ART and TB treatment. Patients newly diagnosed with HIV-1C, not receiving ART, aged 18–45 years old were enrolled during a routine clinical visit prior to starting ART provided informed consent was given. Presence of active TB disease was assessed using a combination of sputum testing using the XPERT MTB/RIF (Cepheid, CA, USA) assay, chest X-ray and clinical history. Immunological markers, CD4 count, and co-receptor tropism were measured cross-sectionally at the enrolment timepoint.

#### Cross-sectional and longitudinal HIV-1B study

Patients enrolled in the prospective Amsterdam Cohort Studies on HIV-1 infection and AIDS among homosexual men (ACS) prior to seroconversion, and with available follow-up prior to and after seroconversion were included, as described elsewhere^53^. Patients were followed up at three-monthly study visits. Immunological marker data, viral load and CD4 count was assessed on samples collected at one year and five years after seroconversion. Viral co-receptor tropism was assessed at three-monthly intervals throughout the study. Antiretroviral treatment was initiated according to the guideline recommendations at that time.

Ethical approval for the cross-sectional HIV-1C study was received from the University of Pretoria Ethics review board and the Limpopo province provincial department of health ethical review board. Ethical approval for the cross-sectional and longitudinal HIV-1B study was received from the Amsterdam University Medical Center institutional ethical committee of the University of Amsterdam. This study was conducted in accordance with the Guidelines for Good Practice in the Conduct of Clinical Trials in Human Participants in South Africa as well as the ethical principles of the World Medical Association Declaration of Helsinki. All study participants provided written informed consent.

### Sampling procedures

For the cross-sectional HIV-1C study, peripheral blood mononuclear cells (PBMCs) were isolated using Ficoll-Hypaque in a LEUCOSEP frit barrier filter tube. For the cross-sectional and longitudinal HIV-1B study, PBMCs were isolated using manual layering without the use of a frit barrier. For both studies, heparin and EDTA-derived plasma were stored at − 80** °**C and cells were viably stored at − 196** °**C in Iscove’s Modified Dulbecco’s Medium (IMDM) with 25 mM HEPES and 1% Penicillin–Streptomycin, 20% Fetal Calf Serum (FCS) and 10% Dimethyl Sulfoxide (DMSO).

### HIV-1 envelope sequence analysis

Nucleic acid extraction was performed on 500 μl EDTA-derived plasma with the NUCLISENS MINIMAG extraction system (miniMAG; bioMerieux, Inc., Durham, NC) as per manufacturer instructions, yielding an eluate of 50 μl. A Reverse-transcriptase-Polymerase Chain Reaction (RT-PCR) was performed in triplicate using the one-step Superscript III Reverse Transcriptase (Invitrogen, CA, USA) on 9 μl of the eluate, using primers covering the complete *env* gene (A1 5′ GGCTTAGGCATCTCCTATGGCAGGAAGAA-3′ and N1 5′-CTGCCAATCAGGGAAGTAGCCTTGTGT-3′). A nested PCR was performed on the pooled RT-PCR products, using the PLATINUM *Taq* DNA Polymerase High Fidelity system (Invitrogen, CA, USA), and the following primers B1 5′-AGAAAGAGCAGAAGACAGTGGCAATGA-3′ and M1 5′-TAGCCCTTCCAGTCCCCCCTTTTCTTTTA-3′^54,55^. Gel electrophoresis was performed on the final PCR product to verify the product length. The product of the nested PCR was cleaned using the GENEJET PCR Purification kit as per manufacturer’s instructions (Thermo Fisher Scientific, Mass, USA).

After preparation of 1 ng input with the Nextera XT DNA Library Preparation kit (Illumina), samples were sequenced using the Miseq (Illumina, San Diego, USA). For indexing the Nextera XT Index Kit v2 Set D was used (Illumina). For normalisation before pooling, the dsDNA concentration was measured with the Qubit ds DNA High Sensitivity (HS) Kit as per manufacturer’s instructions (Life Technologies, Thermo Fisher Scientific Inc.). Paired-End sequencing was done with the MiSeq Reagent v2 kit (500 cycles) (Illumina, MS-102-2003).

### Genotypic assessment of co-receptor usage

In the cross-sectional HIV-1C study, sequences were aligned to the gp160 sequence of a subtype C reference (CISR9292BR025, https://www.hiv.lanl.gov) using the GENIOUS software package (Version 8.1.2). The sequences were extracted and all reads covering the whole V3-region were used in a GTT (geno2pheno[454], G2P-algorithm available at: https://coreceptor.bioinf.mpi-inf.mpg.de/). We reported prediction of X4-tropic virus at a cut-off of  ≤ 3.5% FPR (False-Positive Rate of falsely classifying an R5-tropic virus as an X4-tropic virus), which is commonly used for next generation sequencing data^56–58^.

The top 10% V3-sequences from each patient were processed in the online bioinformatic web-based tool *context-based modelling for expeditious typing (COMET)* for HIV-1 subtyping (https://comet.lih.lu).

### Phenotypical assessment of co-receptor usage

In the cross-sectional and longitudinal HIV-1B study, co-receptor usage of viral strains was assessed three-monthly after seroconversion using phenotypical methods as described elsewhere^59^. Briefly, fresh or cryopreserved PBMCs from HIV-1B infected individuals were co-cultivated with MT-2 lymphoblastoid cells. Isolates producing syncytia in MT-2 cells were considered syncytium-inducing (SI), i.e. X4-tropic viral isolates.

### Measurement of soluble immune activation markers

In the cross-sectional HIV-1C study, immune activation-related soluble proteins were measured in EDTA-plasma using multiplex technology (xMAP; Luminex). The multiplex immunoassay was performed as previously described^60,61^. In short, aspecific heterophilic immunoglobulins were pre-absorbed with HeteroBlock (Omega Chemicals, Hebron, Indiana). The soluble immunological parameters were CCL3 (MIP-1α), CCL4 (MIP-1β), CCL5 (RANTES), CXCL12 (SDF-1α), TNFR2, sCD163, CXCL10 (IP-10) and IL-7 (see Table 2). Measurements were performed with a Bio-Rad FlexMAP3D in combination with xPONENT software version 4.1 (Luminex, Austin, Texas). Data analysis was performed with Bioplex Manager 6.1.1 (BIO-RAD).

### Cell staining and flow cytometric analysis

Half a million PBMCs from the cross-sectional HIV-1C study were thawed, incubated with monoclonal antibodies (see Supplementary Table 1) and fixed using phosphate buffered saline with 0.5% bovine serum albumin, 0.005% Na-Azide and 1% paraformaldehyde. Fluorescence minus one (FMO) controls were used to define positive gates for expression of CD3, CD4, CD8, CD38, HLA-DR, CD70, CD57, CCR5, CXCR4, CD169 (Table 2 and Supplementary Table 1). Lymphocytes and monocytes were gated based on forward and side scatter and expression of the various surface receptors was analysed using a FACS LSR II (BD Biosciences, Franklin Lakes, USA) and FACS Diva software version 7.0 (BD Biosciences, Franklin Lakes, USA). Representative histograms showing the flow cytometry gating strategies are shown in the supplemental Fig. 1.

In the cross-sectional and longitudinal HIV-1B study, PBMCs were sampled every three months. Flowcytometric analysis was performed on available PBMC samples obtained on the timepoint closest to one year after seroconversion as described elsewhere^53^. Briefly, PBMC were thawed and two samples of 0.5 × 10^6^ cells were incubated with monoclonal antibodies for CD4, CD38 and HLA-DR, and fixed using Cellfix. Expression was analysed on a FACSCalibur (BD Biosciences) using Cellquest software.

### Statistical data analyses

In the cross-sectional HIV-1C data, continuous immunological marker data and age were normalised, centred and variables with a skewed distribution were log-transformed. Missing immunological marker data points were imputed using K-nearest neighbor imputation. Exploratory univariate analysis of associations with co-receptor tropism was performed using the unpaired Students t-Test and Mann–Whitney Wilcoxon for continuous data, and χ^2^-test for categorical data. Subsequently, principal component analysis (PCA) was performed on immunological data, TB status, sex and age in order to account for multicollinearity of immunological marker data and distinguish between discrete immunological pathways^62^. Effect of principal components on X4-tropism was estimated using stepwise backward logistic regression with the first four principal components as independent variables and X4-tropism, defined at an FPR cut-off of ≤ 3.5%, as the dichotomous dependent variable (Table 3). Differences were considered statistically significant when p < 0.05.

In order to assess the robustness of the analysis described above and to describe influence from outlier data, both the PCA and the logistic regression analysis were subjected to k-fold cross validation.

Using the longitudinal HIV-1B data, normalizing, centering and log-transformation of data was performed as described for the cross-sectional HIV-1C data. Cross-sectional analysis of HIV-1B at the five year post seroconversion timepoint using the unpaired Students t-Test. The five year timepoint was selected as the majority of tropism switches in the cohort had occurred at this time. Subsequently, the effect of cellular immune activation markers measured one year after seroconversion on subsequent switch in co-receptor usage was assessed using Cox proportional hazard analysis. The one year timepoint was selected as measured immunological markers demonstrate considerable change over time during and after seroconversion. The outcome was defined as switch from R5-tropic to X4-tropic virus using phenotypic assessment of co-receptor usage. Participants in whom a switch was never observed were right-censored at the end of their study follow-up. Cox models were adjusted for age, CD4 count and viral load. Results were reported as crude and adjusted hazard ratios.

## Supplementary information

Supplementary file 1
