# Supplementary material for: Immune activation correlates with and predicts CXCR4 co-receptor tropism switch in HIV-1 infection
Source: Sci Rep. 2020 Sep 28;10:15866. doi: 10.1038/s41598-020-71699-z (PMC7522993; doi:10.1038/s41598-020-71699-z)

**Immune Activation correlates with and predicts CXCR4 co-receptor tropism switch in HIV-1 infection**

Bridgette J Connell^1#^, Lucas Hermans^1,2,3#^, Annemarie M.J. Wensing^1,2,3^, Ingrid Schellens^4^, Pauline J. Schipper^1^, Petra M. van Ham^1^, Dorien T.C.M. de Jong^1^, Sigrid Otto^4^, Tholakele Mathe^3^, Robert Moraba^3^, José A.M. Borghans^4^, Maria A. Papathanasopoulos^5^, Zita Kruize^6^, Francois W.D. Venter^2^, Neeltje A. Kootstra^6^, Hugo Tempelman^3^, Kiki Tesselaar^4^ and Monique Nijhuis^1,2,3*^

^1^Department of Medical Microbiology, Virology, University Medical Center Utrecht (UMCU) The Netherlands, ^2^Ezintsha, Faculty of Health Sciences, University of the Witwatersrand, South Africa, ^3^Ndlovu Research Consortium, Limpopo Province, South Africa, ^4^Center for Translational Immunology, UMCU, ^5^HIV Pathogenesis Research Unit, Faculty of Health Sciences, University of the Witwatersrand, Johannesburg, South Africa. ^6^Amsterdam University Medical Centers, Amsterdam Infection & Immunity Institute, The Netherlands, Academic Medical Center of the University of Amsterdam, Amsterdam, The Netherlands.

***** Corresponding author

E-mail: [m.nijhuis@umcutrecht.nl](mailto:m.nijhuis@umcutrecht.nl) (MN)

T +31 (0)88 75 59558 | F +31 (0)88 7555426

# these authors contributed equally to this work

Running Title: Immune Avtivation and HIV-1 Swtich from R5-X4 tropism


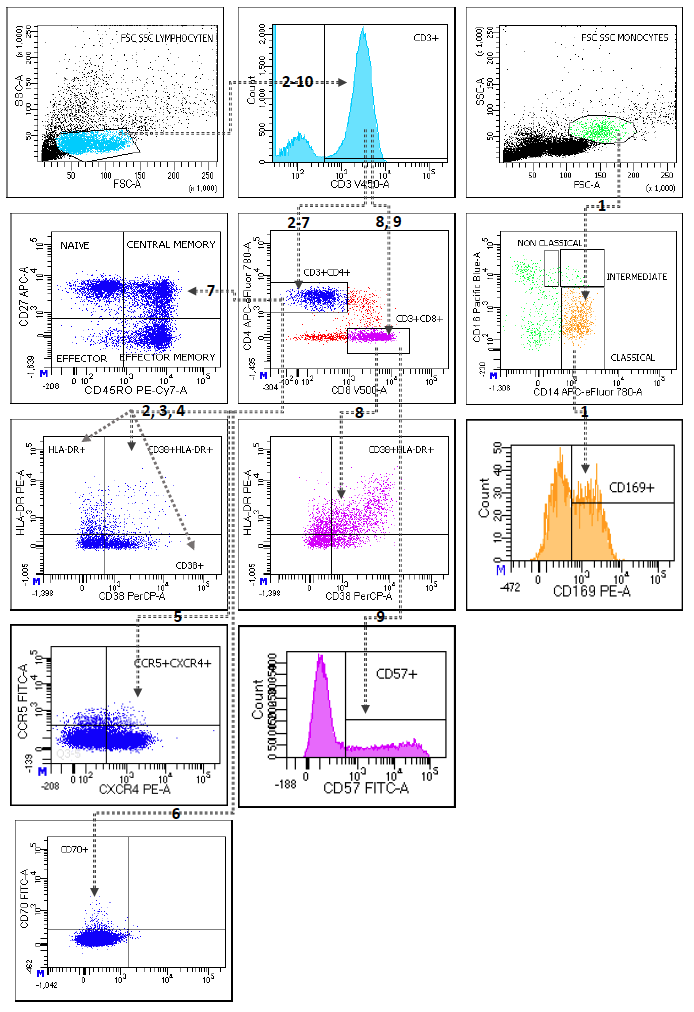


**Supplementary figure 1: Gating strategy for the flow cytometry analysis of immune cells in our analysis. Lymphocytes and monocytes were identified based on  FSC and SSC plots. Within these populations the following percentages were determined. 1)  CD169 expressing classical  monocytes (CD14 high, CD16 dim), 2)  CD38, 3) HLA-Dr , 4) (CD38 and HLA-Dr),  5) CCR5 and CXCR4 6) CD70 or  7) ((CD27 and CD45RO) ,i.e  central memory)) CD3+CD4+expressing  T cells, 8) (CD38+ HLA-Dr+) or 9 ) CD57+ expressing  CD3+CD8+ T cells. The applied strategy is depicted by sequential arrows.**

Supplementary table 1: Staining protocol for flowcytometry in this study

| **Staining** | **T-Cell Activation** | | | | **HIV co-receptors** | | **Monocytes** | |  | |
| --- | --- | --- | --- | --- | --- | --- | --- | --- | --- | --- |
|  | **MoAbs** | **MF** | **MoAbs** | **MF** | **MoAbs** | **MF** | **MoAbs** | **MF** |  |  |
|  | CD3 e450 | eB | CD57 FITC | BD | CD3 e450 | eB | CD169 PE | Ab |  |  |
|  | CD38 PerCp-Cy5.5 | BD |  |  | CD4 APC-Cy7 | eB |  |  |  |  |
|  | CD4 APC | eB |  |  | CXCR4 PE | BC |  |  |  |  |
|  | CD8 v500 | BD |  |  | CCR5 FITC | BD |  |  |  |  |
|  | HLA-DR PE | BD |  |  | CD8 PerCP | BD |  |  |  |  |
|  | CD70 FITC | BD |  |  |  |  |  |  |  |  |
|  |  |  |  |  |  |  |  |  |  |  |
| MoAbs Monoclonal Antibodies MF = manufacturer, eB = eBiosciences (San Diego, U.S.A.), BD = BD Biosciences (Franklin Lakes, U.S.A.) BL BioLegend (San Diego, U.S.A.) Ab = AbD Serotec (Kidlington, U.K.) | | | | | | | | | | |

Supplementary table 2: Variable loadings in principle component analysis


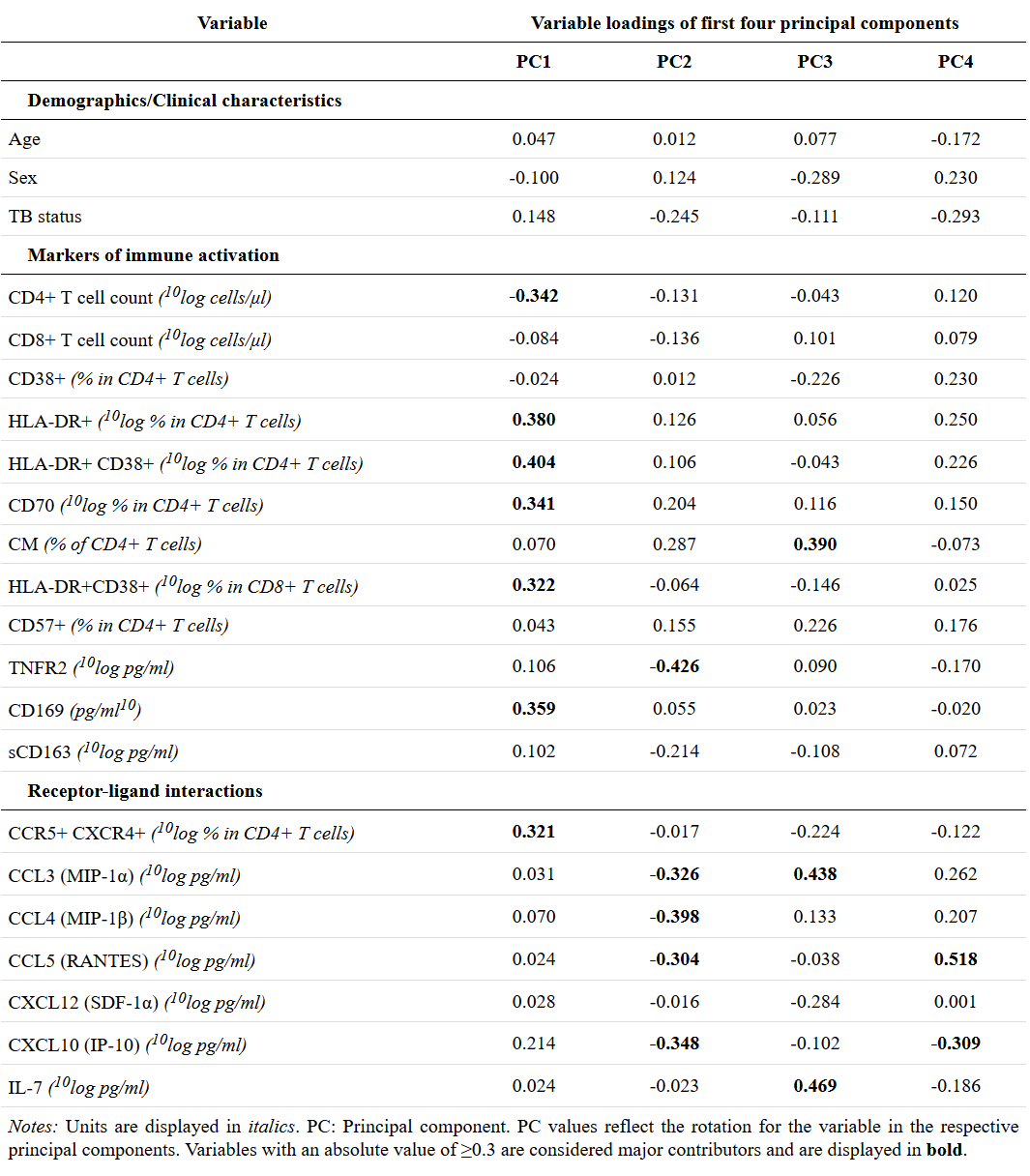


Supplementary table 3: Cross-sectional HIV-1B analysis – Correlates of viral co-receptor tropism at 5 year post seroconversion


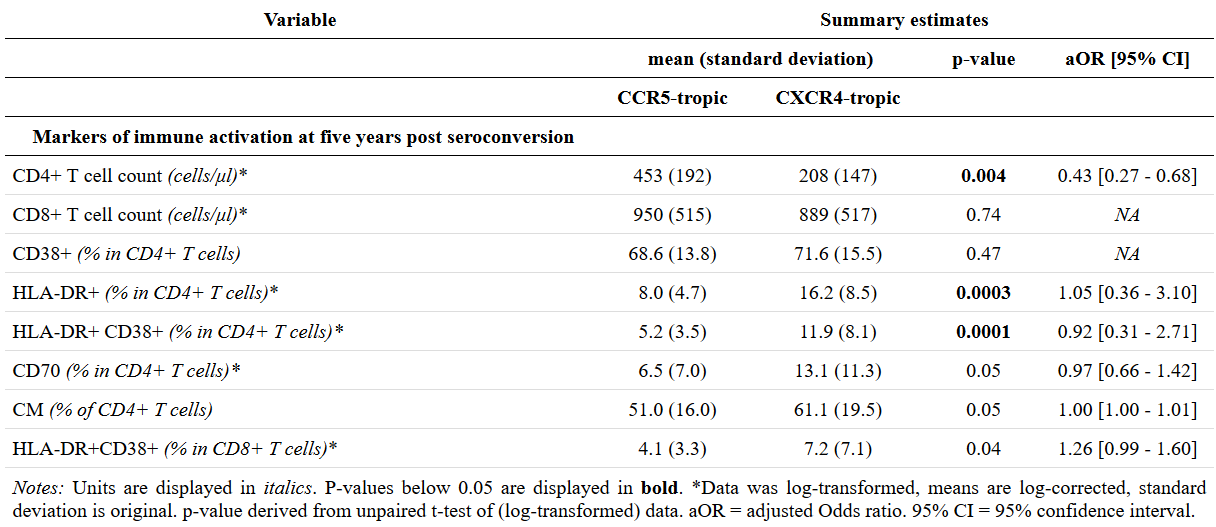

Supplement: Supplementary file 1 [file 41598_2020_71699_MOESM1_ESM.doc]
